# Young Toddlers’ Word Comprehension Is Flexible and Efficient

**DOI:** 10.1371/journal.pone.0073359

**Published:** 2013-08-22

**Authors:** Elika Bergelson, Daniel Swingley

**Affiliations:** 1 Department of Psychology, University of Pennsylvania, Philadelphia, Pennsylvania, United States of America; 2 Institute for Research in Cognitive Science, University of Pennsylvania, Philadelphia, Pennsylvania, United States of America; University of Barcelona, Spain

## Abstract

Much of what is known about word recognition in toddlers comes from eyetracking studies. Here we show that the speed and facility with which children recognize words, as revealed in such studies, cannot be attributed to a task-specific, closed-set strategy; rather, children’s gaze to referents of spoken nouns reflects successful search of the lexicon. Toddlers’ spoken word comprehension was examined in the context of pictures that had two possible names (such as a cup of juice which could be called “cup” or “juice”) and pictures that had only one likely name for toddlers (such as “apple”), using a visual world eye-tracking task and a picture-labeling task (n = 77, mean age, 21 months). Toddlers were just as fast and accurate in fixating named pictures with two likely names as pictures with one. If toddlers do name pictures to themselves, the name provides no apparent benefit in word recognition, because there is no cost to understanding an alternative lexical construal of the picture. In toddlers, as in adults, spoken words rapidly evoke their referents.

## Introduction

“No sooner do we hear the words of a familiar language pronounced in our ears, but the ideas corresponding thereto present themselves to our minds: in the very same instant that sound and the meaning enter the understanding: so closely are they united that it is not in our power to keep out the one, except we exclude the other also. We even act in all respects as if we heard the very thoughts themselves.” G. Berkeley, *An Essay Towards a New Theory of Vision*, Dublin, 1709.

One goal of language science is to understand the interpretation of speech: how the spoken words we hear become ideas in our minds. On current psycholinguistic accounts, listeners integrate the speech signal, their knowledge of the language, and their understanding of the speaker’s likely conversational goals all at once, to arrive at an interpretation of utterances even as the spoken words unfold [1], [2]. As Bishop Berkeley [3] suggested, our facility in understanding language is remarkable, and has given rise to a substantial experimental literature characterizing the cognitive mechanisms at work [4], [5], [6].

The development of spoken language comprehension in children is less well understood, but researchers have claimed that in toddlers, as in adults, interpretation of the speech signal is *incremental* (children attempt to interpret words while the words are being spoken) and, by 24 months, *rapid* (understanding of familiar words in two-year-olds is only a fraction of a second slower than in collegiate adults). These claims rely substantially on procedures in which pictures of potential referents or topics are displayed, and then spoken sentences are presented that either refer to the displayed picture(s) or do not (e.g. [7], [8]). When a spoken word matches the picture a child is looking at, he or she generally continues to look at it; when a word does not match, he or she tends to look away quickly, and in ERP measurements, may manifest the N400 response reflecting a measure of word understanding [7].

Such results are usually interpreted as revealing children’s ability to understand language in general, at least in simple sentences, and not only language in this constrained experimental situation. If this is so, then asking a child about dogs while she is petting the family Weimaraner is, from a speech processing perspective, not fundamentally different from asking her the same question while the dog is out of sight chasing deer. Yet there has been in recent years a series of empirical reports showing the influence of picture presentation on subsequent language processing, raising the possibility that word recognition is strongly affected by the local context. For example, Mani and Plunkett, Experiment 2 [9] found that prior picture presentation could under certain conditions prevent altogether 24-month-olds’ understanding of subsequent words. In the adult literature, Glaser and Glaser [10] found that a related context picture slowed down subsequent target naming (though see [11]).

More generally, numerous studies across the cognitive sciences point to perception under uncertainty as being fundamentally integrative, with interpretive decisions being a product of strikingly diverse sources of information (e.g. [12], [13], [14]). Thus *a priori* there is nothing anomalous or controversial about the possibility that prior picture presentation could affect toddlers’ word recognition, perhaps substantially.

The specific hypothesis we test here is whether recognition of words in object-fixation procedures differs from recognition under more ordinary circumstances by depending primarily on a comparison between the spoken word and the phonological form provoked by viewing an image. We call this hypothesis the *phonological pre-activation hypothesis*. The idea is that children looking at a duck, for example, name it to themselves: “/dˆk/”; and look away from the duck upon hearing a sentence naming anything other than/dˆk/. Clearly this is quite different from ordinary language understanding, in which words give rise to concepts rather than the other way around. If the phonological pre-activation hypothesis is correct, picture fixation procedures as now (widely) implemented would fail to provide appropriate characterizations of word recognition “in the wild” because they rely on a word comprehension mechanism often unavailable in ordinary discourse.

The alternative is that toddlers’ behavior in looking procedures depends upon a semantic comparison. Children hear a word (“book”,/bUk/), leading them to think about books. Then they decide whether the image they are considering is a book or not; if it’s not, they turn their gaze elsewhere. We refer to this hypothesis as the *semantic interpretation hypothesis* [15]. This hypothesis does not say that children or adults are unable to implicitly (or explicitly) name objects they see, but rather that this is not the typical course of word comprehension, and is not necessary for the rapid word understanding shown throughout the psycholinguistic literature and in particular in the large section of this literature that employs eyetracking methods. This hypothesis includes the possibility that children’s language-relevant gaze is affected by semantic priming from the images, a question we address later.

Forms of these two hypotheses have been considered in the literature on word recognition in adults. Tanenhaus and his colleagues, in defending the use of eye-movement or “visual world” procedures to understand language comprehension, have emphasized that listeners fixate objects that share visual features with mentioned referents but that would not be named using the spoken word [16], for instance, looking at a rope when hearing “snake”. They have further shown that language-driven picture fixation is affected by lexical frequency and lexical neighborhood density; if the displayed images were the only ones under consideration, these factors would not impact eye movements. Thus, this set of findings would not be expected if participants were choosing from a small closed set, i.e. if they were phonologically preactivating the labels of the images they see [17]. On the other hand Huettig and his colleagues have stressed that it is difficult to be entirely sure that the presence of visual displays does not make certain concepts or even particular words more available to the listener than they otherwise would have been [18]. These authors do, however, state that the phonological pre-activation hypothesis is incorrect in the adult case (see [19], p. 477).

The research with toddlers has presented a more mixed set of findings. Mani and Plunkett [9], [20], have uncovered some evidence favoring the plausibility of the phonological pre-activation hypothesis. They presented toddlers with one picture in silence, such as an image of a cat. Then, children saw two pictures, one of which was named verbally. On some trials (“*related*” trials), the named target was phonologically related to the prime, as in “cup”, which matches “cat” in the/k/. On “*unrelated”* trials, neither of the two post-prime pictures was phonologically related to the prime image. They found that at 18 months, a related prime facilitated subsequent target looking; at 24 months, it inhibited it. Because the prime was presented in silence, Mani and Plunkett argued that children must have named the prime image to themselves, because otherwise there should be no systematic relationship between the prime picture name’s phonological properties and recognition of the spoken target word. Given results of this sort, it is possible that similar phonological effects apply on test trials in the standard procedure without priming images. One might legitimately suspect on the basis of these results that picture fixation procedures overestimate children’s ability to understand language in more ordinary circumstances. On the other hand, it’s possible that the task employed by Mani and Plunkett puts infants in an atypical situation: perhaps viewing an image in silence, and later having other images labeled creates a visual information processing situation that differs from that in standard picture fixation procedures, and indeed from ordinary word comprehension.

In contrast, Swingley and Fernald [15] provided evidence from a visual-world study supporting the semantic interpretation hypothesis in 24-month-olds. In three experiments, they tested children’s word comprehension in speech referring to present and absent objects and found that regardless of whether the referent was present, children’s eyegaze was conditioned by what they were looking at when they heard the target label: if the spoken word matched the meaning of the gazed-upon object, children maintained their gaze; if it didn’t, regardless of whether the other picture did, they rapidly shifted away. When a nonce word was uttered, children failed to show rapid refixation responses; that is, they did not look away from the image they were looking at quickly. For example, children looking at a ball and hearing “shoe” shifted away from the ball equally quickly when the alternative picture was indeed a shoe, and when it was a car. Furthermore, children looking at a ball and hearing a nonword like “meb” did not refixate quickly. Swingley and Fernald argued that this demonstrated that (a) prior exposure to an image did not measurably impact the speed of recognizing a word for it; and (b) children’s refixation responses must have reflected a search of the lexicon, and not just a test of the match between the spoken word and a word evoked by the fixated picture. This study’s results are not consistent with the phonological preactivation account, which would predict equally fast looks away from an image when hearing a known word, like “shoe”, and a nonce word, like “meb”.

Here, we revisit the question in Swingley and Fernald, with younger children and using methods following an entirely different logic, and without relying on nonsense words. We tested 18–26 month olds rather than 24 month olds, because the second half of the second year is a period in which children vary enormously in the sizes of their vocabularies; studying children over this range permits assessment of potential individual differences in how children treat the task.

A visual fixation procedure was used, which tested children’s word comprehension on two kinds of items: those for which there were at least two viable labels, e.g. a cup of juice which could be called “cup” or “juice”, hereafter *doubles*; and items for which there was only one viable label for toddlers, e.g. “apple”, hereafter *singles*. (Evidence that our characterization of an item as a ‘single’ or ‘double’ matched the children’s is given below.) Singles and doubles were paired in trials, with some children hearing one label for double words and some children hearing the other.

The logic of the study was as follows. With double items, we cannot predict which of the possible names (e.g. “cup” or “juice”) children would use themselves; the label we use in the study would be expected to match the label the children would use themselves approximately half the time. If children pre-name the images they see, and use that word’s phonological form as the basis of their eye gaze behavior as suggested by the phonological pre-activation hypothesis, then about half of the time the word we use will not be the same as the word the child preferred, and children should shift their gaze because of this mismatch. Of course, the other half of the time our target word will be the child’s, and on these trials the *doubles* should not differ from the *singles*. On average, performance should be worse on *double* trials because of the population of trials on which the sound-pattern of the spoken word fails to match the child’s expectations. We confirmed our characterization of the “single-“ or “doublehood” of the pictures by asking children to name the pictures.

In sum, if children’s performance on *double* trials were inferior to their performance on *single* trials, the phonological pre-activation hypothesis would provide a ready explanation. By contrast, if children were to perform equivalently on single and double trials, the results would support the notion that children interpret the sentences meaningfully, and make their eye movement decisions on a semantic basis.

## Methods

### Ethical Statement

All experimental procedures were approved by the IRB of the University of Pennsylvania. All parents provided informed consent in writing on behalf of their infants.

### Participants

Participants were recruited from the Philadelphia area by mail, e-mail, phone, and in person. All children were healthy, carried full-term and heard 75% or more English in the home. None had a history of chronic ear infections. 77 toddlers were included in the final sample (M = 20.95 mo., R = 18.50–26.41 mo., 38 female). An additional five toddlers participated but were excluded due to fussiness (n = 1), equipment failure (n = 3), or failure to meet English exposure requirement (n = 1). Toddlers came from a range of socioeconomic backgrounds, as indexed by mother’s education level gathered by parent report. Participants’ mothers’ educational attainment fell into the following ordered categories: 1) less than high school degree (n = 1), 2) high school diploma (10), 3) some college (8), 4) college degree (16), and 5) advanced degree (41). One parent declined to provide this information.

## Materials

**Figure 1:**
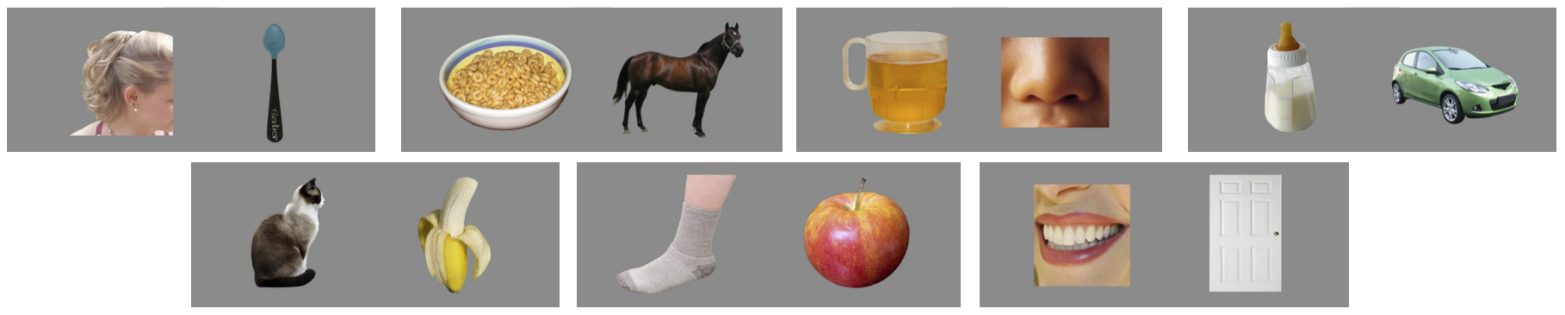
Pairs of images used in the comprehension study. From top left: hair/ear–spoon, cereal/cheerios–horse, cup/juice–nose, bottle/milk–car, kitty/cat–banana, foot–sock/apple, mouth/teeth–door. In the word-comprehension study, infants saw these pairs of images and heard sentences labeling one of the images. In the picture-naming study the images were shown one at a time, and toddlers were encouraged to name them.

Over the course of 28 trials, toddlers were presented with 14 items organized into seven yoked pairs. One member of each pair had two possible labels (doubles, e.g. bottle/milk), and the other only had one likely label to toddlers (singles, e.g. car). (See Table 1 and Figure 1.) Auditory stimuli were pre-recorded sentences, spoken by a native English-speaking woman, and included the target word in one of the following sentence frames: “Can you find the X”, “Do you see the X”, “Where’s the X”, or “Look at the X”. All three target words for a given pair (e.g. bottle/milk, and car) used the same sentence frame. Visual stimuli were photos, displayed side-by-side on a 34.7×26.0-cm LCD screen.

**Table 1:** Details of Experimental Item Pairs.

		Double Label 1	Double Label 2	Single Label
Pair	bottle/milk – car	Bottle	Milk	Car
	cereal/cheerios – horse	Cereal	Cheerios	Horse
	cup/juice – nose	Cup	Juice	Nose
	ear/hair – spoon	Ear	Hair	Spoon
	foot/sock – apple	Foot	Sock	Apple
	kitty/cat – banana	Kitty	Cat	Banana
	mouth/teeth – door	Mouth	Teeth	Door
Measure of frequency	Median 16-mo CDI (understand)	68%	69%	63%
	Median 20-mo CDI (say)	82%	76%	79%
	Median Brent Freq.	249	248	244
	Mean no. mothers using words (of 14)	12	12	13

Experimental item pairs, and measures of the words’ prevalence in children’s vocabularies (Fenson et al., 1994) and in the Brent and Siskind (2001) corpus of infant-directed speech. Each child saw all seven pairs, four times: twice with the single as the target, and twice with the double as the target. Half of the children heard Double Label 1, while the other half heard Double Label 2; no child heard more than one label for the double images.

Each child only heard one label for a given double. Thus, infants saw each of the 7 pairs four times, twice with the ‘single’ image as the target, and twice with the ‘double’ image as the target, but for a given child, only one label was used for the double over the course of the experiment. For instance, Child A would see the pair bottle/milk –car 4 times, twice with ‘car’ as the target and twice with ‘bottle’ as the target; Child B would see the same set of images but hear ‘milk’ instead of hearing ‘bottle’ in the target sentences.

Items were selected and paired in such a way that each set of doubles and the set of singles matched on several measures of frequency, as evaluated in a corpus of 14 mothers speaking to their infants [21], and in a database of parental reports indicating which words parents believed their children understood or said (MCDI) [22]. See Table 1.

### Design and procedure

Toddlers’ visual fixation data were collected using an Eyelink CL computer (SR Research), which provides an average accuracy of 0.5°, sampling from one eye at 500 Hz. It operates using an eye-tracking camera at the bottom of the computer screen; no equipment is mounted on the child’s head, except a small sticker with a high-contrast pattern on it for aiding the eyetracker in keeping the infant’s position.

Before the experiment began, the procedure was explained to parents, informed consent was obtained, and a vocabulary checklist and optional parental background survey were completed. The child and parent were then led to the dimly-lit testing room where the child sat on his or her parent’s lap facing a computer display. Parents wore a visor that prevented them from seeing the screen. On each trial, toddlers saw two side-by-side photos on a grey background. Each trial displayed a single and a double item, one of which was named in a sentence played over computer speakers. Every child heard only one of the double words for a given image, that is, for the pair *bottle/milk–car*, over the course of the experiment Subject 1 heard only *bottle* target sentences, while Subject 2 heard only *milk* target sentences; both heard *car* target sentences. Children saw the pair of images in silence for 2 seconds before the sentence played, and for 3.5s after the sentence played. Sentences varied in length, but were each approximately 1.5–2s long. Every two trials were followed by an attention getting video with flitting shapes and a giggling sound.

After all 28 test trials, children saw each of the 14 pictures used in the study one at a time, and were asked to name them by the experimenter and/or parent, who did not give any clues or information about the meaning or name of the image; in a few cases where such hints were given, those items were removed from analysis. Additionally, the experiment was video-recorded to allow for post-experiment evaluation of the child’s behavior and word production.

All children were randomly assigned to one of two pseudo-randomized trial orders, which counterbalanced label of double item, and side, item-type, and ordering of images and target items. The experiment lasted 15–25 min, after which families were compensated with a choice of $20 or two children’s books. The entire visit lasted about 45 min.

## Results

The analyses laid out below are as follows. First we examine overall performance in the task. Then, we look at the main variable of interest, item-type (singles and doubles) to search for evidence of differentiated performance for single and double trials. We compare these trial-types overall, and in the subset of trials in which infants happened to be looking at the target just before hearing it named (target-initial trials), as these are precisely the trials for which the phonological preactivation and semantic interpretation accounts make the clearest diverging predictions. We then examine whether performance varied the first versus the second time that infants heard a target word named, to search for evidence of naming convergence across trials. We then discuss the children’s own naming of the images after the word-comprehension study. Finally, we create a statistical model of the data to better understand the possible roles of individual-difference variables (mother’s education and child’s age) on the word-comprehension results.

### Baseline Performance

Overall, children showed strong performance in looking at the named target picture. For all analyses we use a time window from 367–1500 ms after the target onset. While this window is a bit shorter than the standard window of 367–2000 ms [23], we chose the shorter window based on our observation that after 1500 ms, toddlers looked away from both the correct and incorrect image at the same rates, suggesting their looking responses were no longer related to the spoken word. Analyses using shorter or longer windows do not change the pattern of results.

The proportion of target looking was significantly above chance (.5) over subjects and items; this pattern held for 63 of 77 subjects and 14 of 14 items (M_subj_ = .63(.013), M_items_ = .65(.080), p<.001 by one-sample Wilcoxon test and binomial test over subjects and over items). (All reported means are followed by standard errors of the mean, and all Wilcoxon tests are two-sided, two-sample paired tests, unless otherwise indicated.) For this and further analyses, the same pattern of significance holds when examining the younger half of the sample or the older half of the sample alone (younger half: n = 39, M_subj_ = .60(.017), M_items_ = .62(.028), older half: n = 38, M_subj_ = .66(.019), M_items_ = .68(.020), all ps<.005 compared to chance); though, as expected, performance correlated with age; Kendall’s tau = .26, p = .001). Because we lack theoretical grounds to separate subjects in this range into age groups, we have elected to maintain one sample, with age as a continuous variable.

The next sets of analyses tested the prediction of the phonological pre-activation account that overall performance on singles would be higher than on doubles, and more specifically, that performance on trials in which toddlers were already fixating the target when the target word was said (target-initial trials) would differ between singles and doubles.

### Singles vs. Doubles Overall

In the window from 367–1500 ms, the proportion of target looking at singles and doubles did not vary (M_single_ = .65(.016), M_double_ = .63 (.016), estimated difference = .013, p = .54 by Wilcoxon test). 42 out of 77 participants had a higher single than double subject means proportion of target looking ; 4/7 pairs showed higher items means for singles than for doubles (both n.s. by binomial and Wilcoxon test).

### Performance on Singles vs. Doubles in Target-Initial Trials

**Figure 2:**
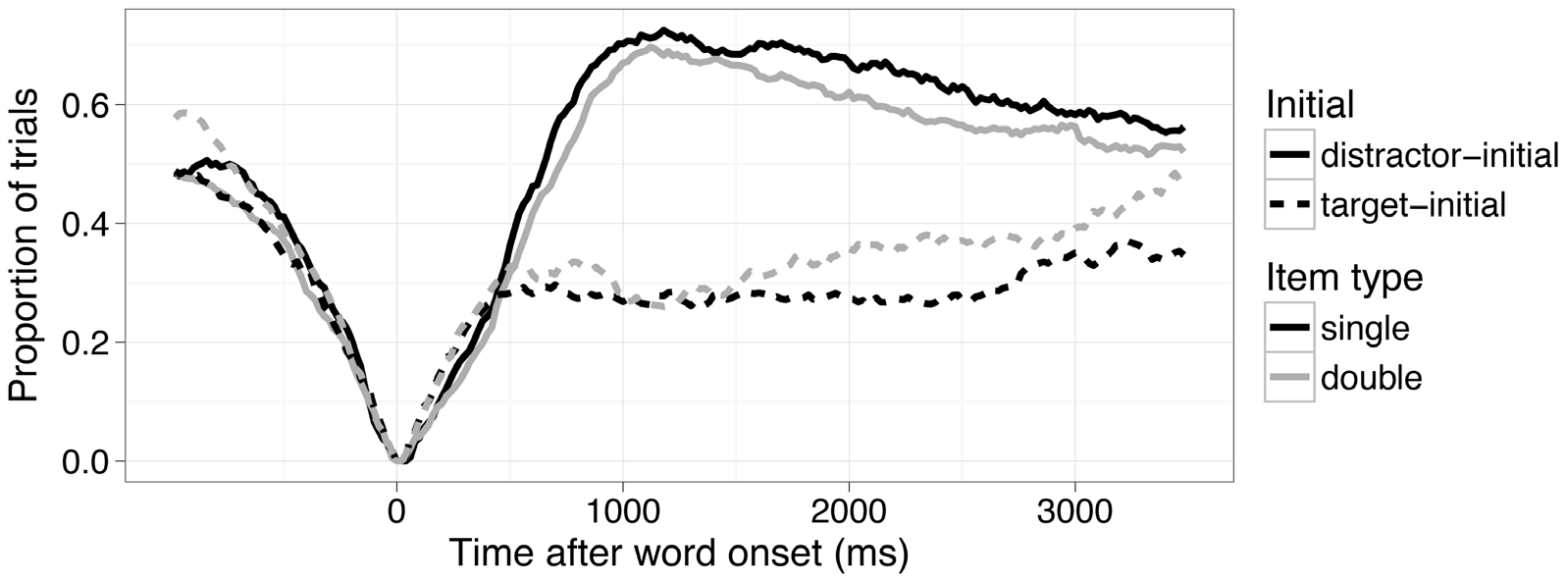
Target fixation, split by initially fixated object and trial-type. This onset-contingent timecourse plot shows, for each unit of time, the proportion of trials in which children are currently fixating an image that is not the one they fixated when the target word began; thus, all trials have a y-value of 0 at time 0. The x-axis shows time, beginning at the onset of the target word. Solid lines show distractor-initial trials, dashed lines show target-initial trials. Grey lines show trials in which doubles are the target, black lines show trials in which singles are the target. On distractor-initial trials, toddlers quickly looked away from the distractor, seen by the fast rise in the solid lines. There is no difference between the single and double performance. In this type of graph, strong performance is demonstrated by the target-initial trials remaining flat near zero, while the distractor-initial trials rapidly rise from 0 towards 1 (See text for further details).

**Figure 3:**
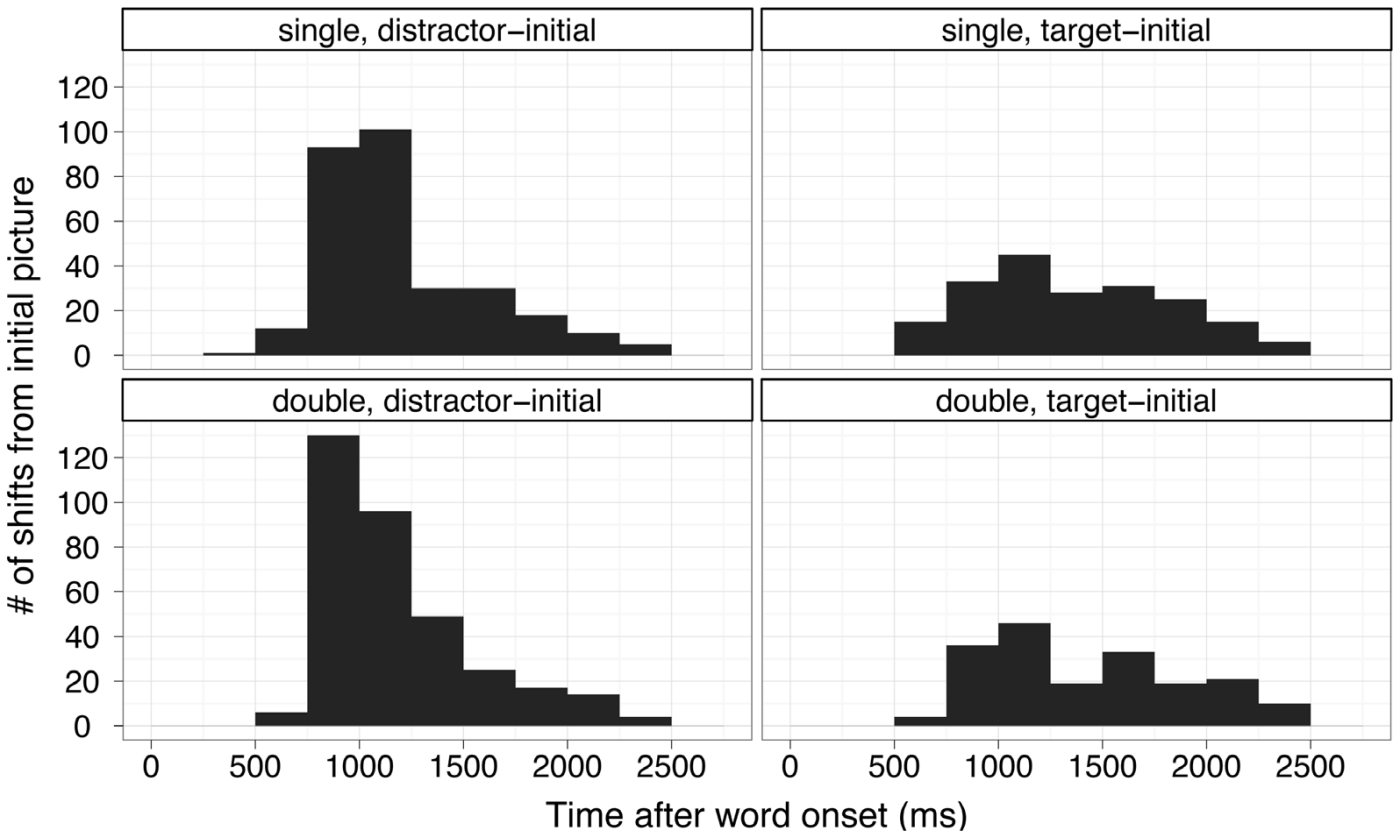
Histogram of refixations between, 367–1500 ms after target onset. Single and double refixations from target and distractor-initial trials, in 250 ms time-bins are shown. Analyses of distributions revealed no significant difference between singles and doubles (all p>.4, see text for details).

Given that the phonological preactivation account predicts that if children do not hear the label they have predicted, they will look away from the currently gazed image, whereas the semantic interpretation hypothesis predicts that infants will maintain their gaze if the incoming label is appropriate for the image based on its meaning, we analyzed the subset of target-initial trials (i.e., those in which infants were looking at the correct image before they heard the sentence) separately. Within these trials we examined the distribution of re-fixations away from the correct picture to the incorrect picture (see Fig. 2&3). We found that over subjects, infants shifted from correct to incorrect pictures at almost identical rates across single and double trials (M_singles_ = .32 (.023), M_doubles_ = .31 (.025); estimated difference = .016, p = .69 by paired Wilcoxon Test). The distributions of these shifts over time did not vary across singles and doubles (D = .10, p = .42 by Kolmogorov-Smirnoff Test over refixation timing; see figure 3, target-initial panels).

We also calculated the proportion of shifts for trials in which parents reported on the vocabulary checklist that their child understands (57% of the data), or understands and says (35% of the data) all three relevant words (e.g. for a cup-juice/nose trials, ‘cup’, ‘juice’, and ‘nose’). The pattern of results in these subsets of the data is the same as those in the overall data: there is no significant difference between the singles and doubles in the proportion of looks away from the target on target-initial trials (estimated difference = .050, p = .55 for ‘understands and says’ trials, estimated difference  = .0047, p = .85 for ‘understands’ trials).

We next computed the proportion of target looking, to examine whether the single/double variable affected the degree of word comprehension (see figure 2). Here, the proportion of target looking again did not differ between singles and doubles (M_target-initial doubles_  = .83(.015), M_target-initial singles_ = .82(.016), estimated difference  = –.0015, p = .93 by Wilcoxon Test). Thus, these analyses provided no support to the phonological-preactivation hypothesis. Children maintained their gaze to the correct picture equally, whether the picture had one name or two potential names.

Because the two accounts do not make differential predictions for the distracter-initial trials (both account predict infants will look away for these trials, as they do, see Figures 2 and 3), we did not analyze those trials separately.

### Singles vs. Doubles in First and Second Instances

Finally, to examine the possibility that children do show phonological pre-activation, but that their preferred label was overridden by the one the experiment provided, we also queried whether toddlers’ responses to singles and doubles varied as a function of whether it was the first trial for a target word or the second (each target word was heard on two separate trials across the experiment; each pair of images appeared 4 times). We found no evidence of differential performance between the first and second instance of the word for singles or for doubles (M_singles, first_ = .63(.018), M_doubles, first_ = .61(.020), M_singles, second_ = .66(.018), M_doubles, second_ = .64(.021), single versus double comparisons for first instance alone and second instance alone showed no significant differences by paired Wilcoxon test; all estimated differences <.02, all ps>.5)

### Word-Labeling Data

Following the test trials, most of the children were willing to name many of the individual pictures they had seen, sometimes requiring some prompting from the parent. Analysis of children’s vocal productions served to validate the item selection by demonstrating that children as a group indeed knew two words for our “double” items and one for our “single” items. On average, children offered an unheard label for the single items less than 1/3 as often as for the double items. We also found that word production increased with age (see table 2). A McNemar test with continuity correction revealed that the number of images that children gave heard vs. unheard labels differed significantly by item type (single vs. double) (Overall: χ^2^(1, N = 694) = 104.76, p<.001; Younger children: χ^2^(1, N = 295) = 42.78, p<.001; Older: χ^2^(1, N = 399) = 61.00, p<.001). This confirms that, at least overall, the double items offered more lexical interpretations than the single items, as the study design required. Furthermore, the words we used for the double images were frequently given alternative names even after children had heard the images labeled twice during the experiment.

**Table 2:** Toddlers’ label production after word comprehension task.

Avg. # of Trials per Subject (out of 7)	Younger Half		Older Half		Overall	
	Single	Double	Single	Double	Single	Double
Child gave unheard label	.38	1.18	.50	1.63	.44	1.40
Child gave heard label	3.46	2.54	4.89	3.47	4.17	3.00
Child said nothing	3.15	3.28	1.61	1.89	2.39	2.60

### Linking Word Comprehension to Later Word Production

We also examined whether the names toddlers provided for each image after the word-comprehension portion of the study predicted the degree to which subjects looked at the target picture. That is, to use a real example, did a child’s calling the mouth/teeth picture “lip-gloss” impact the degree to which she looked at the mouth/teeth picture when it was called “mouth” during the study? In order to analyze this, we took the subset of the data in which toddlers produced a label in the word-production task (694/1078 trials). We then calculated the proportion of target looking in the 367–1500 ms window, for each subject, for each trial, and averaged these values based on whether toddlers produced the heard label or another word, for singles, doubles, and overall. A comparison of these proportions of target looking found no difference in behavior between trials in which children later named the image with the heard label as compared with a different label (M_unheard label_ = .62(.025), M_heard label_ = .65(.012), M_unheard label,singles_  = .58(.048), M_heard label, singles_ = .66(.015), M_unheard label, doubles_ = .63(.029), M_heard label, doubles_ = .64(.019); estimated difference_heard,unheard_  = .023, p = .27 by unpaired Wilcoxon test). There was a trend for better performance in correctly named trials for singles alone (estimated difference.086, p = .094), but not for doubles alone (estimated difference = .00003, p = .95). This seems at least in part a reflection of the rarity of singles labeled with unheard labels overall, e.g. a horse labeled as ‘dog’ by a child. Unheard labels on singles occurred on average.44 times per child out of a possible 7.

Thus, whether children later called an image by the name they had heard in the study or by another name, they were equally successful in looking at the correct picture in the word comprehension portion of the study.

### Modeling Age and Mother’s Education

Given the possibility that individual differences between the subjects were preventing us from seeing underlying effects of item-type (single, double), we modeled the data using mixed-effects models. We modeled the effects of age, productive vocabulary, and mother’s education, along with item-type (single, double), to determine their relative contributions to the proportion of target looking in the 367–1500 ms window (over subject-item means). Using R’s lmer package, we used a maximal linear mixed effect model. The random effects structure included random intercepts for subjects and words, and a random slope for item-type (single/double) grouped by subject. Since a given item could not belong to both item-types, no by-item random slopes for item-type were included. The fixed effects included: total number of our target words infants were reported to say on the MCDI(0–21), age in months (18.5–26.4), residualized by vocabulary, and mother’s education (1–5; see Participants Section). (Age was residualized by vocabulary given that these measures correlated significantly (kendall’s tau = .31, p<.001); analyses indicated that age did not contribute beyond the effect of vocabulary size. That is, including age residualized by vocabulary resulted in a significant contribution of vocabulary alone, while including vocabulary residualized by age resulted in a significant contribution of both.)

**Figure 4:**
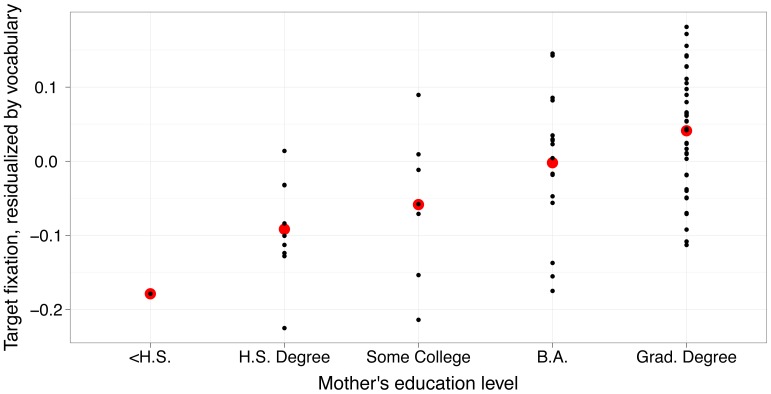
Subject means, by mother’s education level, in 367–2000 ms post-target onset window, residualized by child’s productive vocabulary. Each small black dot represents a single subjects’ mean performance over items. The larger red dot indicates the mean for that education level, residualized by vocabulary. A model including vocabulary and mother’s education shows a 4.9% increase in performance for each level of education.

Vocabulary size and mother’s education were found to be significant predictors of toddlers’ proportion of target looking; the model estimated that target looking increased.67% per word (out of 21) and 4.9% per mother’s-education category (T>4 & p<.01 by log likelihood ratio test for both predictors; see table 3 & figure 4). (As a perhaps more intuitive measure, a model including age instead of productive vocabulary found a 5.3% performance increase per education level, and a 1.5% performance increase per month of age.) In sum, infants’ target-looking in a model taking into account individual differences did not indicate any difference between performance on singles versus doubles.

**Table 3:** Model of Subject-Item Means.

Model Predictors	Estimate	SE	T value	P value
Age residualized by Vocabulary	.0055	.0062	.879	1.00
Productive Vocabulary (0–21)	.0067	.0014	2.49**	.0061**
Mother’s Education (1–5)	.049	.0092	4.71**	<.0001**
Item Type (Single/Double)	–.011	.044	–.26	1.00

Estimates, standard errors, T values, from the model, and P values estimated by chi square log likelihood ratio test for each predictor are shown. This model also included random intercepts for subjects and words, and a random slope for item-type (single/double) grouped by subject.

We also ran analogous logistic multilevel models by converting subject-item means to 0s and 1s based on whether they were above or below.5. While this simplifies the data, it obviates any risks due to the potentially peculiar variance structure of proportional data [24]. The results of this model paralleled those presented above: item-type was not a significant predictor of toddlers’ looking behavior.

## Discussion

This study shows that before their second birthday, toddlers’ word comprehension is flexible and efficient. Upon hearing a word, toddlers are able to link the incoming word-form to its meaning, and use this information to guide their attention to an appropriate visual referent for the word; they do this equally well whether the pictured referent is name-ambiguous (double items), or not (single items). This pattern was found both in overall target-looking, and in target-initial trials’ pattern of fixations away from the target.

We suggest that these results are incompatible with the phonological pre-activation hypothesis: were infants to have activated the names of the pictures they were looking at before the target was uttered and used these names to guide their behavior, we would have found different patterns of eye movements to single and double items. In several different analyses, we found no such evidence, lending credence to the semantic interpretation hypothesis.

The phonological pre-activation account predicts that a child who pre-named an image “cup” should look away from the cup-image upon hearing “juice” because this does not match the sounds the child expected. Thus, on trials in which the child was already fixating the target image (target-initial trials), for doubles, this hypothesis predicts that about half the time children would be expected to look away, because these target-initial trials are, effectively, distractor-initial trials for that child. We can estimate the size of this predicted effect using the target- and distractor-initial single trials: by 1.5s after the target word began, toddlers looked away from the correct picture 31.5% of the time and away from the incorrect picture 54.9% of the time. Under the phonological pre-activation hypothesis, children are equally likely to be in each of these states on the double trials, yielding the prediction that children should look away from the target on approximately (0.5 * (31.5+54.9)  =  ) 43.2% of target-initial trials, viz. a 11.7% increase in shifts from their performance on target-initial single trials. No such increase is present in children’s behavior. Target-initial fixation behavior is similar on double and single trials: infants shift away from the correct picture on 31.1% of target-initial double trials, and the distribution of their shifts is virtually identical across singles and doubles. We found a.4% decrease in shifts away from the target for doubles, rather than the 11.7% increase predicted by the phonological pre-activation account (see Results).

Of course, a null effect cannot guarantee that a cognitive phenomenon does not exist. But our evidence does show that if children do base their decisions at least partially on their phonological expectations, it must provide a very small contribution to their behavior, rather than the large effect predicted by the phonological activation account. More concretely, we can estimate the effect size predicted by that account, and compare it to the effect size we find. In the present study the phonological pre-activation account predicts an effect of size 0.51 (M_estimated from singles_ = .117, SD_estimated from singles_ = .23). In fact, the observed effect size was a nonsignificant 0.018 in the opposite direction (M_computed from doubles_ = –.0037, SD_computed from doubles_ = .21). Thus, while the phonological preactivation account would predict that toddlers’ behavior in target-initial trials would resemble the average of their behavior on target- and distractor-initial single trials, we find no evidence in support of this; in contrast, the pattern of results is entirely consistent with the semantic interpretation hypothesis.

One possibility that these data leave open is that phonological preactivation is a mechanism called upon in certain experimental paradigms, in which, for instance, single images are shown in isolation with no clear pragmatic or task context (as in Mani and Plunkett [9]). That is, it is possible that when infants are shown a single image, in silence, followed by a pair of images, that this might change how infants view the task, causing them to engage in a range of processes that may not be present in the more standard language-guided looking method. For instance, it is possible that the ongoing speech stream, instantiated here as a carrier phrase, may disrupt any covert naming that might otherwise occur with silently presented images, or that viewing multiple images creates an environment of competition for attention, which annuls processes that may occur in a free-viewing, single-image context; we thank an anonymous reviewer for suggestions along these lines. The pattern of results here, however, suggests that in the more common language-guided-looking tasks used with infants (a subset of the visual-world tasks used with adults), no such mechanism is recruited and that instead, flexible, rapid, semantic interpretation occurs. This is in keeping with the adult spoken-word comprehension studies by Tanenhaus and colleagues, and with the Swingley and Fernald work with older toddlers (see Introduction). We leave open to future work a consolidation between our results with a multiple-referent visual world and those of Mani & Plunkett [9], and colleagues, which may perhaps uncover the use of different mechanisms in different word-comprehension tasks or contexts.

Additionally, we found no evidence of any age-related effects due to item-type, but rather a significant, gradual improvement with age over our nine-month range. This confirms to us that age was best treated as a continuous variable, but even when dichotomized into an older and younger group, our results showed the same patterns. Similarly, as others have documented, [25], [26], [27], mother’s education has a significant and graded effect on toddlers’ overall word-comprehension performance. These effects were large, as Figure 4 shows. Our data do not speak to the origins of performance differences correlated with socioeconomic status; based on the work of previous authors, differences in the children’s language environment are plausible causes.

While our findings do not offer support for the phonological pre-activation hypothesis, there remains the possibility, compatible with the semantic interpretation hypothesis, that semantic priming from the pictures impacted children’s language-relevant eye movements. That is, the speed and efficiency with which words were recognized in this experiment may have been due to the potent influence of the images to conjure up the words’ meanings [28]. If this were the case, then our results, among others, would tell us something only about a “constrained referent” case of word comprehension, rather than “true” word comprehension, as it is often manifest in daily language use.

We have two reasons to doubt that semantic priming from the images is responsible for the effects we find. First, Swingley & Fernald [15] found that when toddlers were looking at the wrong picture, they switched away equally fast when the other available picture was the target as when it was an unrelated distracter image that they were familiar with. This suggests that toddlers are in a way performing a ‘go/no go’ task in which they continue to look at a picture until they have evidence that it is not the correct image (i.e. hearing a label that doesn’t apply to the picture), at which point they rapidly shift to another image, even if the other image is also inconsistent with the word they heard. Semantic priming from the images would not support this pattern. Similarly, in the current data set, if we analyze just the subset of trials on which infants had only looked at the distracter image before hearing the target word, but saw both images by the end of the trial, their level of performance was above chance, and did not vary statistically from their performance on trials in which they had seen only the target image or both images prior to hearing the target image labeled (M = .68, p<.001 by one-sample Wilcoxon Test; estimated difference from other trials<.001, p = .83 by Wilcoxon Test.) Thus, even when subjects hadn’t seen the target picture yet, they quickly shifted their gaze to it, and away from the incompatible distracter image. A semantic priming account would have predicted higher levels of performance on trials in which the target image had been seen prior to the label being heard; this is not what we found.

Casting doubt on the “phonological pre-activation” hypothesis in children as young as 19 months should come as happy news for experimentalists who use visual fixation procedures, but the importance of this fact goes beyond support for the validity of prior studies using such methods. The fact that 19 month olds rapidly grasp words’ meanings even without significant contextual support is consistent with the real-world ecology of language use between parents and children. Although parents talk about the “here and now” with their children to a greater degree than do adults speaking among themselves, a significant proportion of parental word usages occur in the absence of the object or event referred to [29]. Moreover, by around 15 months, young children are able to track adults’ references to absent objects to some degree; this ability becomes more robust by around 22 months when toddlers can update their mental representation of absent objects [30], [31], [32].

Several lines of evidence suggest that both facility in rapid word recognition and skills of absent-referent language understanding contribute to the acquisition of language. Speed and accuracy of word recognition in the language-guided looking task at 25 months of age predict expressive vocabulary growth in the second year [33], and linguistic and cognitive skills at age 8 [34]. Quick translation of the acoustic signal into a mental model of an utterance’s meaning may relieve demands on working memory and allow for greater attention to interpretation of the utterance’s syntax and its function in the discourse, and may enhance children’s capacity for isolating the referents of novel words [35]. Likewise, the ability to identify nouns in sentences fosters learning of verbs [36]. This process occurs in two-year olds even when children hear sentences with no concrete referential context whatever [37], [38].

In conclusion, our findings lend support to the semantic interpretation hypothesis. Rather than rigidly committing to predicted phonological forms upon seeing images, ambiguous or otherwise, our data suggest that by around 20 months of age, children comprehend words flexibly and efficiently, much as adults do. They can do this even when it requires them to construe referential contexts in more than one way, as in the case of pictures that could be *bottle* or *milk*, *mouth* or *teeth*. Parents’ utterances take a perspective that often differs from the child’s, and this need not hinder children’s comprehension. Our findings underscore the flexible power of children’s treatment of spoken language: words present ideas to children’s minds [3].
